# Supplementary material for: Piezoresistive Sensor Based on Micrographite-Glass Thick Films
Source: Sensors (Basel). 2022 Apr 24;22(9):3256. doi: 10.3390/s22093256 (PMC9100864; doi:10.3390/s22093256)
Supplement: Supplementary file 1 [file sensors-22-03256-s001.zip › sensors-1638887-supplementary.pdf]

New composite material based on micrographite particles in glassy matrices for applications in piezoresistive sensors

Osvaldo Corrêa
Center for Semiconductor Components and Nanotechnologies
University of Campinas
Campinas, Brazil
https://orcid.org/0000-0002-8619-2239

Pompeu Pereira de Abreu Filho
Center for Semiconductor Components and Nanotechnologies
University of Campinas
Campinas, Brazil
https://orcid.org/0000-0001-5248-6098

Mara Adriana Canesqui
Center for Semiconductor Components and Nanotechnologies
University of Campinas
Campinas, Brazil
macanesq@unicamp.br

Stanislav Moshkalev
Center for Semiconductor Components and Nanotechnologies
University of Campinas
Campinas, Brazil
stanisla@unicamp.br

Jacobus Willibrordus Swart
School of Electric and Computer Enginnering
University of Campinas
Campinas, Brazil
jacobus@fee.unicamp.br

Abstract—**New composite piezoresistive material has been developed based on micrographite particles immersed in glassy matrix with composition containing several Pb-free oxides: Bi_2O_3, B_2O_3, SiO_2, Al_2O_3 and ZnO. This composition allowed to obtain reduced sintering temperature for the composite material thus avoiding loss of graphite during thermal processing in air. Methodology for producing piezoresistive films from pastes with micrographite particles includes the use of a sodium carboxymethyl cellulose (NaCMC) aqueous solutions during preparation of pastes. It was verified that NaCMC plays a decisive role in interactions between graphite particles and glassy matrix, providing good wettability of glassy matrix particles and homogeneous distribution of micrographite particles in the pastes. The films stability and piezoresistive characteristics of the films were verified in the mechanical tension experiments.**

Keywords—piezoresistive material, thick film, conductive paste, micrographite particles, glassy matrix.

I. Introduction

The piezoresistive thick film technology was introduced decades ago for production of hybrid circuits, resistors and components for various industrial segments, such as automotive, military, aerospace, information technology, computing and instrumentation [1 - 3]. This technology is based on functional phases of ruthenium oxide and / or lead (or bismuth) ruthenates. This is an area where most of R&D results are confidential and belong to military and industrial sectors. Thus, the literature discussing the applications and production of electronic devices with thick films (thickness between 40 to 100 µm) is scarce. What the publications have been showing are devices made in a laboratory scale with the intention (not always really confirmed) of being industrialized.

Currently, the typical process of piezoresistive films fabrication for hybrid circuits is based on three basic elements: i) a functional phase of conductive oxide particles, such as RuO_2, $Bi_2Ru_2O_7$ or $Pb_2Ru_2O_6$; ii) a glassy matrix (permanent binder) consisting of glasses, such as Pb or Bi borosilicates and iii) an organic phase (vehicle) composed of a volatile phase (for example, α-terpineol) and a non-volatile phase (ethyl cellulose polymer). In addition, small amounts of dispersants and plasticizers are present to improve homogeneity, rheology of pastes and facilitate printability (screen-printing) on substrates. The thick films obtained after thermal processing (between 800 and 850°C) are mounted on devices having their electrical resistances adjusted to the desired values by restricting (cutting) small areas of the films by a laser equipment (Laser Trimming System) [4].

The piezoresistive film fabrication process presented in this article uses graphite micron scale particles obtained by grinding of natural graphite to replace ruthenium oxides. The glassy matrix consists of a system without the presence of Pb, and the organic (non-volatile) phase is an aqueous solution of sodium carboxymethyl cellulose (NaCMC). The final temperature of the thermal processing (sintering) was maintained at 600°C to prevent the losses of graphite by oxidation in air. This article represents the preliminary results of the research with pastes containing micrographite particles.

It is important to emphasize that this research is original, the bibliographic search of references in this area for recent years did not show any articles and patents related to piezoresistive films based on micrographite particles for sensor applications.

The replacement of ruthenium oxide by micro graphite was justified considering the price and low availability of metallic Ru (Ru^0). Ru^0 is a platinum group metal (8B), not very abundant in the earth's crust [5]. The positive demand for ruthenium has kept its price between US $ 6,430 / kg and US $ 11,254 / kg, with the South Africa being the largest producer supplying ~ 90% of the world consumption [6, 7]. The use of ruthenium oxides had been justified by their thermal stability, resistance to oxidation and electro migration. Even considering that the commercialized pastes have an average of 15 to 20% of ruthenium oxides, their prices are highly impacted by the costs of Ru^0. For example, the DP (DuPont) 2401 paste had been quoted at US $ 8,700 / kg. The shortage of Ru^0 also does not encourage industrial R&D investments in products based in Ru^0. Therefore, besides the much lower cost and higher availability, micro graphite has other significant advantages, such as thermal resistance, high conductivity and absence of electro migration at high temperatures. Micrographite used in the present work, is commercialized by

XXX-X-XXXX-XXXX-X/XX/$XX.00 ©20XX IEEE

Nacional de Grafite (Itapacerica, MG), under the name Micrograf 99503UJ.

II. Experimental

A. Materials

The lateral size of micrographite particles Micrograf 99503UJ used here is close to 3 µm, and the mean thickness is in the order of magnitude smaller (100-200 nm). The tapped density of the graphite powder is 0.21 g/cm^3. The sodium carboxy methyl cellulose (NaCMC) was supplied by Denver, Cotia, SP. with pH=6.9 (4% solution) and viscosity coefficient = 90 cP(4% solution, 25°C). The other analytical grade reagents were: Bi_2O_3, ZnO, SiO_2, Al_2O_3, H_3BO_3, Ag / Pd conductive Paste DuPont 7484R and alumina plates with dimensions of 5 x 2.5 cm and thickness of 730 µm.

B. Preparation of glassy matrix

The preparation of glassy matrix (composed by Bi_2O_3, B_2O_3, SiO_2, Al_2O_3 and ZnO) was done under ambient conditions by mixing of glass components. The solid mixture of reagents was homogenized and transferred to an alumina crucible. Then, the crucible was transferred to an oven at a room temperature and heated to 900°C with a rate of 10°C / minute and 10 minutes at final temperature. After melting, the liquid mixture was poured quickly into cool deionized water following a grinding in a mortar grinder Retsch. After the grinding, the glassy matrix was sieved through a 200-mesh sieve (74 µm). The tapped density of the sieved glassy matrix ($\leqslant$ 74 µm) was 3.80 g/cm^3.

C. Preparation of conductive film of Ag / Pd

The conductive film Ag / Pd was deposited on the alumina plates to form conductive tracks for electrical contacts of Micrograf 99503UJ conductive film. This film was made using the DuPont 7484R paste applied on alumina plate in presence of masks made with a tape (thickness ~ 125 µm) to control the thickness and position of film. After application of DuPont 7484R paste, the deposited films were kept in rest for ~ 15 min. and afterwards the masks were removed. Then, the films were heated initially at 250°C (heating speed 10°C / min., and 10 minutes at a final temperature) and after reaching 850°C maintaining the same conditions of the first heating. This thermal processing was carried out using an oven EDG, model FCVI - I. For electrical contacts, copper wires (AWG 23) were welded with Sn / Pb weld (63/37) over Ag / Pd tracks (Figure 1A).

D. Preparation of conductive paste

The conductive graphite based paste was prepared using solid mixtures with a mass relation for micrographite:glassy matrix of 1:10 adding the aqueous solution of sodium carboxymethyl cellulose (NaCMC) of 1.83% (w /w). The mass relation of Micrograf 99503UJ: NaCMC in the paste was between 1: 0.06 and 1: 0.09. The mixtures, after adding the NaCMC solution, were homogenized and then evenly distributed with a squeegee over alumina substrates.

E. Preparation of the conducting films

The micrographite based films were made by depositing the paste described above on alumina substrates with dimensions of 5 x 2.5 cm and thickness of 700 µm. Before deposition, masks were prepared on the alumina substrate with a tape (thickness ~ 125 µm) to mark the position and width (5 mm) of the film. After resting about 15 minutes at ambient conditions, the masks were removed, and the films were heated up to 600°C (rate of 10°C /minute and 10 minutes at final temperature) in air.

F. Determination of electrical resistence of conductive films under mechanical tension (flexure)

Piezoresistive responses (ΔR) of the material to mechanical tension (flexure) were evaluated for films with parameters as follows: length ~ 50 mm, width ~ 5 mm and thicknesses typically between 80 and 150 µm (Fig. 1A). The ends of the conductive film were deposited over Ag / Pd film tracks that served as a conductive path, and the Cu wires (AWG 23) were welded on the film track to enable electrical contacts in electrical measurements using a multi-meter Agilent 34401A.

The piezoresistivity response was checked using a cantilever clamped beam geometry. In this approach, the beam flexure was generated by a calibrated force (load) perpendicular to its base, applied at the free (not clamped) end of the cantilever. In this situation, the change of the film electric resistance under flexure and corresponding film deformation together with the alumina substrate is due to changes in the contacts between conductive graphite particles in the film. These changes result in increase of the detected electrical resistance.

The mechanisms of electrical conduction in the piezoresistive films based on micrographite particles are still not well understood and need further studies. There is some discussion in the literature for other systems with conductive particles of different origin, including RuO_2, which shows that the standard percolation theory of transport universality was proved only in a limited number of experiments on real disordered composites [8]. In these experiments, the tunneling-percolation model was applied to carbon-poly(vinylchloride) composites where the inter-grain tunneling was found to be the principal mechanism of transport [9]. An attempt to develop a theoretical model to relate the resulting gauge factors in piezoresistive sensors to different types of nanofillers has been reported in a recent work [10]. It is thus possible to suggest that for the micrographite based piezoresistive films a tunneling-percolation model is also valid.

The clamped beam experiments were done using the set up showed in Fig. 1B. In the measurements of the electrical resistances during loads, the samples were kept under action of each load for 30 seconds. After the removal of each load, there was an interval of 2 minutes and then the next load was performed.

III. Exterimental results and discussions

In most experiments, the best micrographite to glassy matrix mass relation, where better adhesion of the films to alumina substrates was observed, was 1:10 with the volume relation glassy matrix to micrografite of 0.6: 1. The presence of NaCMC was necessary for formation of homogeneous pastes with adequate viscosities for spreading on alumina substrates. The adhesion was tested by a manual force applied with a spatula at the interface film/substrate. The strong adhesion made impossible the film detachment. Another evidence of strong film adhesion and hardness was obtained in the film abrasion tests with sandpapers.

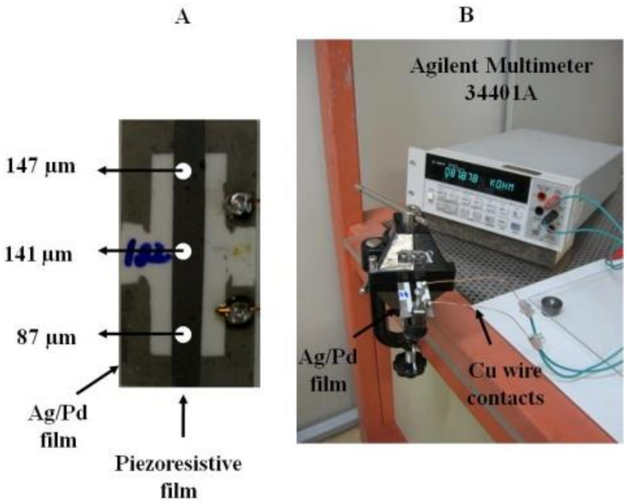

Fig. 1. (A) Resistor sample. (B) Experimental set up for measurements of electrical resistance under load in the clamped alumina substrate

In the absence of NaCMC and with a paste containing only graphite filler, glassy matrix and water, the formation of a continuous film with good adherence to the substrate was not possible. This result shows that NaCMC has a decisive role in the interactions between micrographite particles and glassy matrix. The NaCMC chains dissolved in water are amphiphilic, and they establish van der Waals bonds with the initially hydrophobic surfaces of micrographite particles. This results in deep modification of graphitic surfaces providing their hydrophilicity due to hydroxyl groups of NaCMC chains bonded to the graphitic surface. Probably, partial intercalation of micrographite particles by NaCMC molecules followed by their exfoliation also occurs in these interactions. These hydrophilic micrografite-NaCMC composite particles are responsible for wettability of the resulting pastes and good adherence to the alumina substrates.

The results of electrical measurements under and after flexure experiments with the load force applied up to 800 g are shown in Fig. 2, where the piezoresistive effect for prepared conductive film (with the film resistivity of 2.0 Ω.cm) can be observed with increase in the electrical resistance under action of loads. Almost linear increase of the film resistance with the load was verified. Reductions in electrical resistances after load removal to the initial levels were also determined, with these measurements being made after two minutes of removal of each load. Another important result was the absence of an abrupt increase in the electric resistance or loss of conductivity during the loading caused by irreversible microcracks in the structure. Note that preliminary experiments were performed with linear configuration of deformation sensors. Better sensitivity for the mechanical deformations is obtained in sensors based on the Wheatstone bridge configuration, these experiments are now in progress.

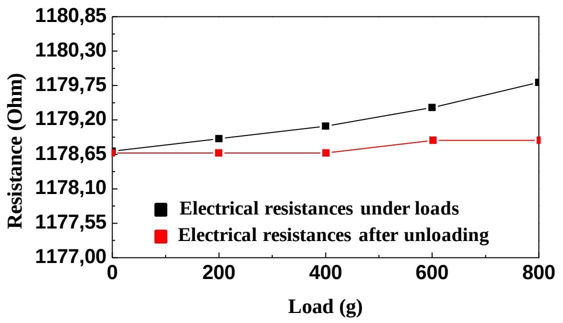

Fig. 2. Electrical resistance of the piezoresistive film under loading and unloading

The scanning electron microscopy (SEM) images were obtained using a Dual beam FIB/SEMNova 200 Nanolab. The figure 3 shows the SEM image of the film surface where micrographite particles (grey color) are uniformly distributed in the glassy matrix. Some pores (darker areas) in the film surface can be also seen. The brighter areas correspond to non-conductive glassy matrix particles that are charged by the electron beam.

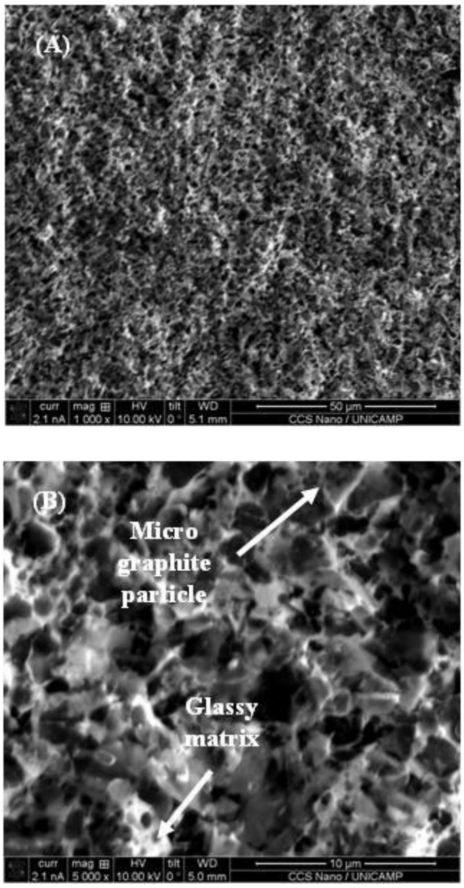

Fig. 3. Scanning electronic microscopy image of piezoresistive film surface of sample 102 (Figure 1 A), Scale bars 50 µm (A) and 10 µm (B

Figure 4 shows a cross-section of the fractured film, with lower (left) and higher (right) magnifications. Uniform distribution of micrographite particles in the glassy matrix and the presence of some pores can be also observed.

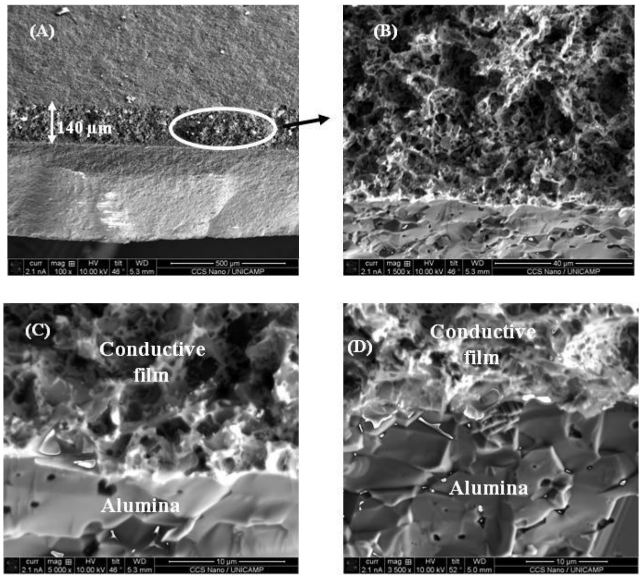

Fig. 4. Scanning electronic microscopy image of piezoresistive film cross-section fracture surface (A e B)) and interface conductive film / Alumina (C e D). Scale bars 500 µm (A) , 40µm (B), 10 µm (C) and (D).

IV. Conclusions

In summary, several conclusions can be made as results of the present work:

i) Using Pb-free glass with composition containing several oxides (Bi_2O_3, B_2O_3, SiO_2, Al_2O_3 and ZnO), it was possible to produce piezoresistive films without loss of micrographite, and with good adherence on alumina substrates attributed in part to the reactions of ZnO and Bi_2O_3 with alumina substrates probably forming $ZnAl_2O_4$ and $BiAlO_3$ at the alumina / film interface;

ii) The 1:10 micrographite: glassy matrix mass relation was found to be optimal to enhance the adherence of piezoresistive films on alumina substrates;

iii) The piezoresistive characteristics of the films were verified in the mechanical tension experiments. These results showed high durability of the films, with full restauration of the film resistance after load removal.

iv) The use of sodium carboxymethyl cellulose (NaCMC) with its amphiphilic character was important to establish van der Waals bonds with micrographite particles transforming their hydrophobic surfaces in hydrophilic. This results in formation of composite particles that could be dispersed in aqueous medium and promote high wettability of glass matrix particles and homogeneity of pastes.

v) The role of NaCMC was decisive in obtaining high quality piezoresistive films obtained with micro graphite. It was verified that pastes obtained in the absence of CMC, did not show good adherence and were not stable when deposited over alumina substrates.

Acknowledgments

The authors thank the CCSNano staff for technical assistance and Dr. A. Alaferdov for SEM images. The authors also thank CNPq, FAPESP and FINEP for financial support.

References

[1] M. N. White and J. D. Turner. Thick-film sensors: Past, present and future. Measurement Science and Technology, vol. 8, pp 1-20, 1997

[2] L. J. Golonka, B. W. Licznerski, K. Nitsch and Teterycz, H. Thick-film humidity sensors. Measurement Science and Technology, vol.8, pp 92-8, 1997.

[3] S. P. Beeby. Printed thick-film mechanical microsystems (MEMS). Materials Science and Applications in Sensors, Electronics and Photonics. Woodhead Publishing Series in Electronic and Optical Materials, pp 259-277, 2012.

[4] R. E. Coté. The effect of high speed laser trimming on accuracy and stability of thick film resistors. Electrocomponent Science and Technology,vol 8, pp181-7, 1981.

[5] Rare Earth Elements - Critical Resources for High Technology https://pubs.usgs.gov/fs/2002/fs087-02/. Accessed em 01/04/21.

[6] PGM Market report. February 2019. Johnson Mathey. http://www.platinum.matthey.com/documents/new-item/pgm%20market%20reports/pgm_market_report_february_2019.pdf. Accessed em 01/04/21.

[7] Heraeus Precious Forecast, 6 February, 2019. https://www.heraeus.com/media/media/hpm/doc_hpm/precious_metal_update/forecast_1/HPM_EM_Forecast_EN.pdf. Accessed em 01/04/21.

[8] S. Vionnet-Menot, C. Grimaldi, T. Maeder, S. Strässler and P. Ryser. Tunneling-percolation origin of nonuniversality: Theory and experiments. Phtsical Review B, 71, 064201, 2005.

[9] E. K. Sichel, j. I. Gittleman and P. Sheng. Transport properties of composite material carbon-poly(vynil chloride). Physical Review B, 18(10), 5712-16, 1978.

[10] M. Bragaglia, L. Paleari, F. R. Lamastra ,D. Puglia, F. Fabbrocino and F. Nanni. Graphene nanoplatelet, nanoplatelet epoxy nano composites as strain sensing coatings. Journal of Reinforced Plastics and Composites,Vol. 40, 1–12, 2021, (Article in press). DOI: 10.1177/0731684421994324
